# Mindfulness based interventions in multiple sclerosis - a systematic review

**DOI:** 10.1186/1471-2377-14-15

**Published:** 2014-01-17

**Authors:** Robert Simpson, Jo Booth, Maggie Lawrence, Sharon Byrne, Frances Mair, Stewart Mercer

**Affiliations:** 1 General Practice and Primary Care, Institute of Health and Wellbeing, University of Glasgow, Glasgow, G12 9LX, Scotland, UK; 2 Institute for Applied Health Research/School of Health and Life Sciences, Glasgow Caledonian University, Glasgow, G4 0BA, Scotland, UK

**Keywords:** Multiple sclerosis, Mindfulness, Stress management

## Abstract

**Background:**

Multiple sclerosis (MS) is a stressful condition; depression, anxiety, pain and fatigue are all common problems. Mindfulness based interventions (MBIs) mitigate stress and prevent relapse in depression and are increasingly being used in healthcare. However, there are currently no systematic reviews of MBIs in people with MS. This review aims to evaluate the effectiveness of MBIs in people with MS.

**Methods:**

Systematic searches were carried out in seven major databases, using both subject headings and key words. Papers were screened, data extracted, quality appraised, and analysed by two reviewers independently, using predefined criteria. Study quality was assessed using the Cochrane Collaboration risk of bias tool. Perceived stress was the primary outcome. Secondary outcomes include mental health, physical health, quality of life, and health service utilisation. Statistical meta-analysis was not possible. Disagreements were adjudicated by a third party reviewer.

**Results:**

Three studies (n = 183 participants) were included in the final analysis. The studies were undertaken in Wales (n = 16, randomised controlled trial - (RCT)), Switzerland (n = 150, RCT), and the United States (n = 17, controlled trial). 146 (80%) participants were female; mean age (SD) was 48.6 (9.4) years. Relapsing remitting MS was the main diagnostic category (n = 123, 67%); 43 (26%) had secondary progressive disease; and the remainder were unspecified. MBIs lasted 6–8 weeks; attrition rates were variable (5-43%); all employed pre- post- measures; two had longer follow up; one at 3, and one at 6 months. Socio-economic status of participants was not made explicit; health service utilisation and costs were not reported. No study reported on perceived stress. All studies reported quality of life (QOL), mental health (anxiety and depression), physical (fatigue, standing balance, pain), and psychosocial measures. Statistically significant beneficial effects relating to QOL, mental health, and selected physical health measures were sustained at 3- and 6- month follow up.

**Conclusion:**

From the limited data available, MBIs may benefit some MS patients in terms of QOL, mental health, and some physical health measures. Further studies are needed to clarify how MBIs might best serve the MS population.

## Background

Multiple sclerosis (MS) is a chronic, unpredictable, and poorly understood neurodegenerative inflammatory condition [1]. Nervous system damage can be extensive, with severe disability in both physical and cognitive realms [2,3]. Worldwide incidence of MS is increasing, with estimates at 3.6/100,000 person-years in females and 2.0/100,000 person-years in men [4]. MS can present in myriad different ways and carries a high degree of uncertainty, in terms of disease progression and resultant impairment [5-7]. MS characteristically falls into several different diagnostic subclassifications, depending on disease activity and stage of progression [8,9].

MS typically follows a chronic and eventually progressive course. Consequently, health service utilisation costs accumulate and impact significantly on resource allocation [10]. Epidemiological data from the United States, Canada, and China all highlight comorbidity as problematic [11-14], with mental health diagnoses frequently co-existing. The literature demonstrates anxiety and depression point prevalence estimates of up to 16.5% and 46%, respectively [15]. Health related quality of life (HRQOL) is often significantly impaired in people with MS [16], and may be exacerbated by disease uncertainty [5] and depression [13]. Mental health comorbidity is thought to be under-reported in people with MS [15,17] and is associated with diminished treatment adherence, increased somatic symptoms, and impairment of both functional ability, and social status [18]. As in many chronic illnesses, self-efficacy is of importance in people with MS. Enhanced feelings of control and acceptance may lessen the psychological and emotional impact of living with a chronic condition, and contribute to improvements in clinical status, such as diminished fatigue [19,20].

Psychological distress may contribute to MS disease activity [21], and a growing body of research evidence examines this hypothesis [22,23]. Cognitive behavioural therapy (CBT) has already been found to impact positively upon psychological stress [24] and pathological neuroimaging markers; the short term effect size for such CBT interventions has been shown in one study to rival that of certain disease modifying pharmacological agents [22].

### Mindfulness based interventions

Mindfulness practices originate from ancient Buddhist meditation techniques, but have since been secularised, manualised, and appropriated for use in a diverse range of clinical settings. Jon Kabat-Zinn, who introduced Mindfulness techniques to the West in the nineteen eighties, has defined Mindfulness as: ‘..paying attention in a particular way: on purpose, in the present moment, and nonjudgementally’ [25]. The original research studies on Mindfulness focussed on chronic pain, but Mindfulness-based interventions (MBIs) have become increasingly popular in various areas of chronic disease management over the last 30 years. Group-oriented, Mindfulness Based Stress Reduction (MBSR) is the most well researched MBI approach and has been applied in the management of: anxiety, chronic pain, depression, and stroke [26-29]. MBSR appears to have neuroendocrine, immunological and neuroplastic effects, although research in this area remains explorative in nature [30-33]. MBSR classically consists of instruction in three meditation techniques, namely breath awareness, body awareness, and dynamic yoga postures (mindful movement) [34] taught in groups over 8 weeks. Mindfulness Based Cognitive Therapy (MBCT) is a derivative of MBSR, with a greater emphasis on cognitive techniques, designed for specific mental health conditions such as recurrent depression [35].

### Why is it important to undertake a review of MBIs?

The mitigation of stress is proposed as a means to actively manage and reduce pathological disease activity in people with MS [22]. Indeed, a CBT intervention has already demonstrated efficacy, reducing gadolinium lesion enhancement on neuroimaging during the active period of treatment. However, this effect is not sustained on cessation of therapist input [22]. MBIs are thought to operate in a different manner to CBT [36] and might have a more sustained effect, given the strong emphasis on regular self-practice. To date and as far as we are aware, no systematic review of the evidence for MBIs in people with MS has been published. This paper aims to evaluate the effectiveness of MBIs in people with MS.

## Methods

### Search strategy

In May 2013 a systematic search for published and unpublished studies was conducted in six major electronic bibliographic databases: Cochrane Central Register of Controlled Trials, MEDLINE, EMBASE, CINAHL, Allied and Complementary Medicine Database, and PsycInfo. To identify any additional published and/or unpublished trials, we also searched ProQuest Dissertations & Theses Database and contacted MS/mindfulness researchers. Selected medical subject headings were combined with key words relating to MS and mindfulness to create a search strategy which was finalised for use in MEDLINE (see Additional file 1) and amended for use in the other databases, using appropriate controlled vocabulary, Boolean operators, and search symbols. Delimiters were: dates searched (1980–2013); research subjects (human); and language (English). The search included the grey literature, using reference lists and citation searching from reviews and published trials, the Science Citation Index, and also involved consulting noted experts in the field. Endnote was used to store and manage the results of the database searches.

### Selection criteria

Studies were selected for inclusion using criteria related to the Study design, Participants, Interventions, and Outcomes model (SPIO). SPIO is an adapted version of PICOs (Population, Interventions, Comparison, and Outcomes) [37]. Any definite diagnosis of MS in an adult (>18 years) was acceptable for inclusion in the review. MBIs can vary by both name and range of ingredients; therefore a core content of: breath awareness, body awareness, and mindful movement, comparable to that of the standardized MBSR, was decided upon as a pre-requisite for inclusion. MBSR was chosen as it represents the original model introduced by Jon Kabat-Zinn in the nineteen eighties, which has been widely described and researched since its inception. Perceived stress was the primary outcome measure sought. Secondary outcome measures included: mental health, physical health, psychosocial measures, as well as health care utilisation.

### Selection of papers for inclusion

The bibliographic records identified by the searches were screened for relevance using broad inclusion criteria, i.e. ‘multiple sclerosis’ and ‘mindfulness’. All relevant papers were then screened, using the SPIO inclusion criteria (Table 1), to select eligible papers. All selected papers were subject to methodological appraisal. As the search yielded low numbers, a decision was made not to exclude studies solely on the basis of poor methodological quality. Screening methods were based on the Systematic Reviews guidance outlined in the Centre for Reviews and Dissemination (CRD) [38]. Methodological issues are discussed below and reported in the evidence table (Table 2).

**Table 1:** **SPIO narrow screen inclusion**/**exclusion criteria**

	**Inclusion criteria**	**Exclusion criteria**
**Study design**	Randomised controlled trial, controlled trial	Qualitative studies Single case study Systematic reviews Literature reviews Guidelines Audit
**Population**	Age >18 years Any diagnosis of MS	<18 years old Diseases other than (and not including) MS
**Intervention**	Any specifically mindfulness-based intervention (MBI)	Psychotherapy Drug treatments Manual therapy (ie massage)
**Outcomes**	Perceived stress Anxiety Depression HRQOL Pain Personal wellbeing Social participation	

**Table 2:** Study characteristics

**Study (country)**	**Study design (setting)**	**Sample size (attrition %)**	**Type of intervention (duration)**	**Outcome measures**	**Data collection**
**Mills and Allen **[41]** (Wales)**	RCT (Patients home)	n = 16 (12.5%)	Mindful breathing Mindful movement (Tai Chi) Self compassion Home study material (6/52 duration)	POMS Standing balance Symptom rating questionnaire	Baseline
					Post intervention
					3 months post intervention
**Grossman et al. **[40]** (Switzerland)**	RCT (University hospital)	n = 150 (5%)	Mindful breathing Mindful movement (Yoga) Body scan Home study material (8/52 duration)	CES-DSTAI MFIS HAQUAMS PQOLC Neuropsych.	Baseline
					Post intervention
					6 months post intervention
**Tavee et al. **[42]** (United States)**	CT (University hospital)	n = 17 (43%)	Mindful breathing (Samatha) Mindful movement (Tai Chi) Walking meditation (8/52 duration)	SF-36 MFIS VAS Physical role Vitality PDDS	Baseline
					Post intervention
					NR

1. RCT - Randomised controlled trial; 2. CT - Controlled trial; 3. CES-D Center for epidemiological studies depression scale ; 4. HAQUAMS - Hamburg quality of life questionnaire in multiple sclerosis (German); 5. MFIS - Modified fatigue impact scale; 6. POMS - Profile of mood states; 7. PQOLC - Profile of health related quality of life in chronic disorders (German); 8. SF-36 - Short form 36; 9. STAI - Spielberger trait anxiety inventory; 10. VAS - Visual analogue scale for bodily pain; 11. PDDS - Patient Determined Disease Steps; 12. Neuropsych. - Neuropsychological assessment; 13. NR - not recorded.

### Quality appraisal

Risk of bias was assessed using the Cochrane Collaboration’s assessment tool [39] to summarise the risk of bias for major outcomes. The evidence for each individual outcome was graded as low, unclear, or high risk. This included assessing for evidence of: sequence generation; allocation concealment; blinding of participants, personnel and outcome assessors; incomplete outcome data; selective outcome reporting; and any other sources of bias.

### Data extraction

The authors developed a data extraction tool, adapted from a previous systematic review examining the benefits of MBIs following transient ischaemic attack and stroke [29]. The data extracted included information on study design and methodology, the populations under review, the interventions being employed, and the outcomes recorded in each study. Two reviewers working independently carried out screening and data extraction. Broad screening was undertaken by RS and SB; narrow screening by RS and JB. Any disagreements were adjudicated via a further reviewer (SM).

### Data synthesis

As the results of the search and review were heterogenous, findings are presented in a narrative format. It was not possible to undertake a meta-analysis.

## Results

**Figure 1:**
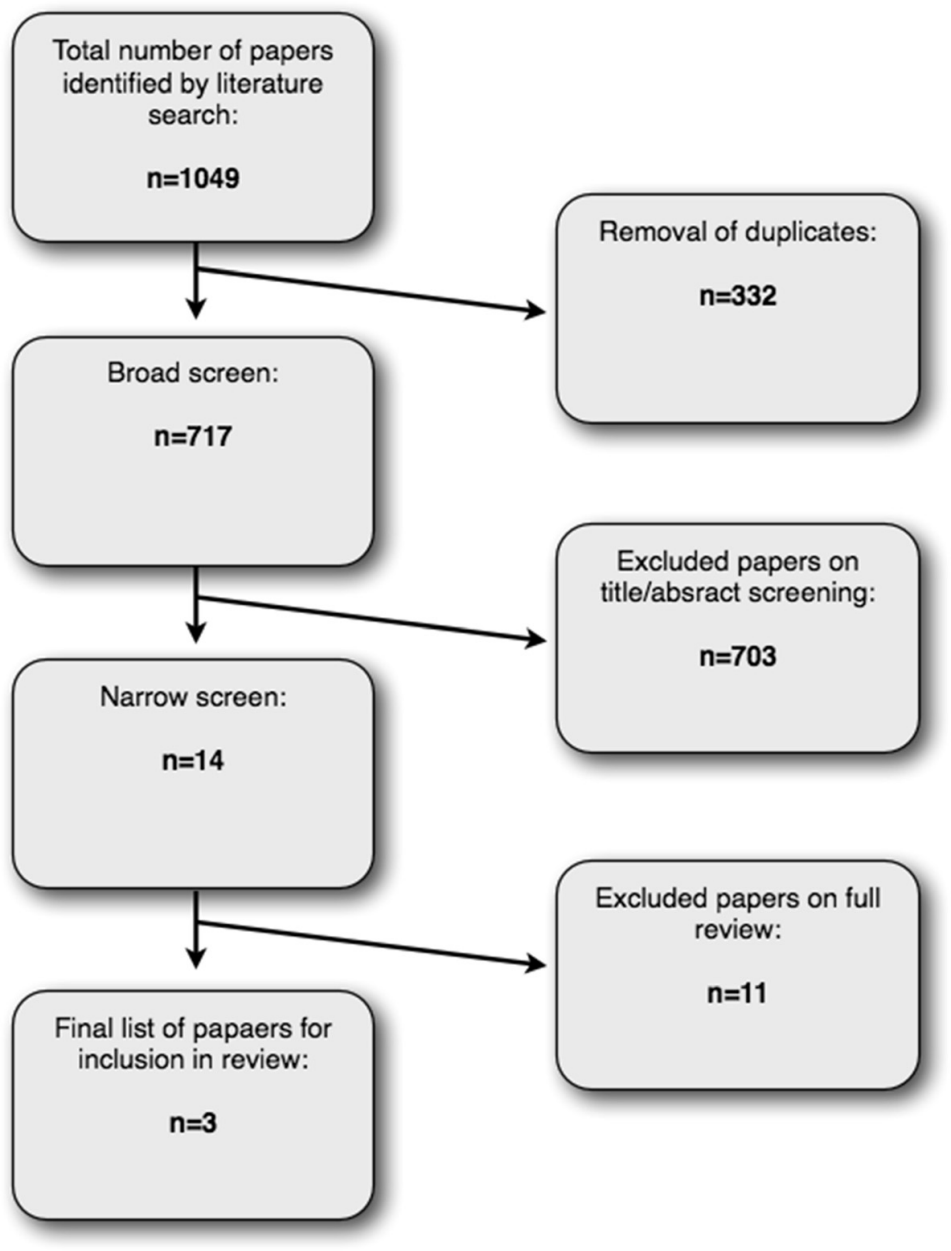
Search results flow diagram.

The search of the databases retrieved 1,049 records. Following screening (Figure 1), 3 papers were considered eligible for inclusion in the review: Grossman et al. [40], Mills and Allen [41], and Tavee et al. [42]. See Additional file 2 for details of papers excluded by narrow screening. Further information on study findings was sought from authors Mills and Allen [41] and Tavee et al. [42], but no responses were received.

### Study characteristics

The three studies originated from Wales (Mills and Allen [41]), Switzerland (Grossman et al. [40]), and the United States (Tavee et al. [42]). Grossman et al. [40] and Mills and Allen [41] conducted randomised controlled trials (RCT), while Tavee et al. [42] reported a non-randomised controlled trial. None of the studies compared MBIs against an active intervention. Grossman et al. [40] recruited from Neurology out-patients, and conducted the study in a university hospital setting, as did Tavee et al. [42]. Mills and Allen [41] recruited from a mixture of General Practitioner and Physiotherapist referrals.

Across the studies, there was a total of 183 participants. Attrition was variable. Grossman et al. [40] described remarkably low attrition rates (5%) and a high attendance rate (92%), whilst Mills and Allen [41] reported drop out rates of 12.5% and cited reasons such as bereavement and dislike of Tai Chi style exercises. Tavee et al. [42] had the highest attrition of all (43%), and cited issues such as transportation and a lack of interest.

Tavee et al. [42] presented results for a mixed study population i.e. people with MS and patients with Peripheral Neuropathy. As we were unable to obtain MS-only data, this limited interpretation of their findings. Grossman et al. [40] collected data at 3 points (pre- and post- intervention, and at 6-month follow up), while Mills and Allen [41] recorded data at pre-, post- and 3 months after the intervention. Tavee et al. [42] only recorded data at pre- and immediately post-intervention. See Table 2 for details.

### Intervention characteristics

Grossman et al. [40] delivered an intervention closely mapped to MBSR. Mills and Allen [41] and Tavee et al. [42] used less standardised versions, introducing elements of Tai Chi and Qi Gong. However, all three studies were broadly comparable in content, with all three emphasising mindful breath awareness, mindful movement, and body awareness or ‘scanning’. The MBIs were group based in 2 of the studies, but the Mills and Allen [41] intervention was delivered one to one.

Grossman et al. [40] used certified and experienced MBI teachers to deliver the MBI over 8 weekly 2.5 hour sessions, with a 7 hour session at week 6. They emphasised mindfulness practices in the sitting, lying and yoga asana postures, and also encouraged ‘homework’ practices for 40 minutes daily. They were the only investigators to record homework practice times. Tavee et al. [42] delivered a 4 hour introductory group session, followed by 8 weekly 1.5 hour classes, with a Buddhist Monk teaching all of the course components, including Samatha meditation (sitting and observing the breath), and mindful movement in the form of Tai Chi/Qi Gong and walking meditation. Tavee et al. [42] encouraged home practice, but did not record frequencies. Mills and Allen [41] delivered six individualised sessions in which unspecified teachers taught mindful movement with Tai Chi/Qi Gong, with breath and posture awareness, whilst encouraging participants to cultivate compassionate feelings towards themselves. Mills and Allen [41] also provided self study material, but did not report on participant uptake and usage by participants. Please see Table 2.

### Participant characteristics

None of the studies provided data regarding ethnicity of participants. Across the three studies, 80% (n = 146) of participants were female, and the mean (SD) age of the total sample (n = 183) was 48.6 (9.4) years. Data regarding socioeconomic status was generally not well reported; for example, Mills and Allen [41] provided data on only half of their study population, of whom, 50% were in employment. Grossman et al. [40] recorded number of years in education. Disease phenotype was described in two of the studies; however, Tavee et al. [42] simply described participants (n = 17) as having any diagnosis of MS. From the remaining 166 patients where a phenotype could be discerned, 123 (67%) had a diagnosis of relapsing remitting MS, and the other 43 (25%) were diagnosed with secondary progressive MS. All three studies excluded patients with significant cognitive impairment, as well as those with severe physical disability, according to either the Expanded Disability Status Scale being >6 (requiring 2 walking aids i.e. a pair of canes, crutches, etc. - in order to walk about 20 meters, without resting), or more generally, being unable to make their own way to the hospital (Mills and Allen [41]). See Table 3.

**Table 3:** Participant characteristics

	**Mills and Allen **[41]	**Grossman et al. **[40]	**Tavee et al. **[42]
**Ethnicity**	NR	NR	NR
**Number of participants (% female)**	16 (80%)	150 (80%)	17 (78%)
**Mean age (SD)**	49.8 (6.8)	47.3 (10.3)	48.7 (11.2)
**Socio-economic status**	NR	NR	NR
**Employment status**	4 employed (25%)	NR	NR
**Mean years of education (SD)**	NR	14.1 (1.9)	NR
**Disease phenotype**	SP 16 (100%)	RR 123 (82%) SP 27 (18%)	NR
**Stage in disease progression**	NR	Mean EDSS 3.0 (1.1)	Mean EDSS 3.0 (2.5)
**Comorbidities**	NR	NR	NR
**Number of patients on disease modifying medication**	NR	91 (60.1%)	NR
**Number of patients on psychotropic medication**	NR	30 (20%)	NR

1. SP - Secondary Progressive; 2. RR - Relapsing Remitting; 3. EDSS - Expanded disability status scale; 4. NR - Not recorded.

### Outcomes

The primary outcome sought by this review (perceived stress) was not reported in any of the studies. However, secondary outcomes of interest included: anxiety, depression, HRQOL, concentration, fatigue, vitality and general wellbeing. No information was available on economic parameters, such as cost/benefit analysis for patients, or health service utilisation. Please see Table 2.

#### Mental health outcomes

#### Anxiety

Grossman et al. [40] measured anxiety directly with the Spielberger Trait Anxiety Index (STAI), demonstrating significant reduction immediately post completion in both whole intervention group and in further subgroup analyses of those with evidence of pre-intervention impairment. This was maintained at six-month follow up in overall group and subgroup analyses. Mills and Allen [41] included data on change in anxiety scores via a general MS symptom checklist, and the Profile of Mood States (POMS), reporting non-significant change. Please refer to Table 4.

**Table 4:** Mental health outcomes

**Study**		**Outcome (measure)**	**Post intervention effect size (p)**	**Follow up effect size (p) and time point**
Grossman et al. [40]	Full intervention group	Anxiety (STAI)	0.39 (0.0006)	0.36 (0.02) at six months
	Sub-group analysis		1.00 (0.002)	0.64 (0.05) at six months
	Full intervention group	Depression (CES-D)	0.65 (0.00001)	0.36 (0.03) at six months
	Sub-group analysis		1.06 (0.0002)	0.66 (0.03) at six months
Mills and Allen [41]		Anxiety (POMS)	p > 0.05*	p > 0.05*
		Depression (POMS)	p < 0.01*	NR

1. STAI - Spielberger Trait Anxiety Index; 2. CES-D - Centre for Epidemiological Studies Depression Scale; 3. POMS - Profile of Mood States.

*Effect size not recorded.

#### Depression

Two studies assessed the effect of MBIs on depression. Grossman et al. [40] used the Center for Epidemiological Studies Depression (CES-D) scale, reporting significant reductions in both whole intervention group and subgroup analyses of those with pre-intervention impairment at intervention completion. This was maintained at six-month follow up in the overall group and in subgroup analyses. Mills and Allen [41] also reported a significant change using the POMS scale. See Table 4.

#### Physical outcomes

#### Standing balance

Mills and Allen [41] reported preferentially on physical measures, opting to focus on single-leg standing balance, with significant improvement noted at both study completion and at three-month follow up, although one participant’s data was missing for this latter calculation. Please see Table 5.

**Table 5:** Physical outcomes

**Study**		**Outcome (measure)**	**Post intervention effect size (p)**	**Follow up effect size (p) and time point**
Grossman et al. [40]	Full intervention group	Fatigue (MFIS)	0.41 (0.0001)	0.38 (0.001) at six months
	Sub-group analysis		1.27 (0.0005)	1.09 (0.02) at six months
Mills and Allen [41]		Fatigue (POMS)	p > 0.05*	NR
		Single leg standing balance	p < 0.05*	p < 0.05* at three months
Tavee et al. [42]		Fatigue (MFIS)	p = 0.035*	NR
		Pain (VAS)	p = 0.031*	NR
		PDDS	p > 0.05*	NR

1. MFIS - Modified Fatigue Index Scale; 2. POMS - Profile of Mood States; 3. VAS - Visual Analogue Scale for pain; 4. PDDS - Patient Determined Disease Steps; 5. NR - Not recorded; *Effect size not recorded.

#### Pain

At study completion, Tavee et al. [42] described a significant reduction in bodily pain, as measured by the Visual Analogue Scale (VAS). See Table 5.

#### Fatigue

All three studies measured the effect of MBIs on fatigue. Scores on the Modified Fatigue Impact Scale (MFIS) were significantly reduced in the study by Grossman et al. [40] at both intervention completion in the overall population, as well as in subgroup analyses of those with pre-intervention impairment. Beneficial effect was maintained in the overall group at six-months, as well as in those with pre-intervention impairment. Tavee et al. [42] also reported significant change on MFIS at study completion. Fatigue was non-significantly reduced on POMS in the Mills and Allen [41] study see Table 5.

#### Psychosocial outcomes

Grossman et al. [40] measured both disease-specific (Hamburg Quality of Life Questionnaire in Multiple Sclerosis; HAQUAMS) and generic HRQOL (Profile of Health-Related Quality of Life in Chronic Disorders; PQOLC), with PQOLC being significantly improved at study completion in the overall group and in subgroup analyses for those with pre-intervention impairment, as well as at six-month follow up in overall group and in subgroup analyses. Similarly, HAQUAMS was significantly improved at completion in the overall group and in subgroup analyses in those with pre-intervention impairment, as well as at six-month follow up in overall group and subgroup analyses. Mills and Allen [41] report an overall trend towards general symptom improvement, but did not justify this statistically. Tavee et al. [42] did not report population-specific results for the Short Form-36 (SF-36). See Table 6.

**Table 6:** Quality of life outcomes

**Study**		**Measure**	**Post intervention effect size (p)**	**Follow up effect size (p) and time point**
Grossman et al. [40]	Full intervention group	HAQUAMS	0.43 (0.0002)	0.28 (0.04) at six months
		PQOLC	0.86 (0.00000001)	0.51 (0.03) at six months
	Sub-group analysis	HAQUAMS	1.01 (0.0001)	0.58 (0.04) at six months
		PQOLC	1.71 (0.00000001)	0.51 (0.003) at six months

1. HAQUAMS - Hamburg Quality of Life Questionnaire in Multiple Sclerosis (German); 2. PQOLC - Profile of Health-related Quality of Life in chronic disorders (German).

### Methodological quality of included papers

Quality was assessed using the Cochrane Collaboration tool for Risk of Bias [39]. Of the 3 studies, only Grossman et al. [40] adequately describe evidence of sequence generation at the randomisation stage. Allocation concealment was most convincingly implemented by Grossman et al. [40], where the investigator was fully blinded to patient information, but this was not clearly described by Mills and Allen [41]; Tavee et al. [42] appear to have collected a control group independently of those expressing a desire to take part in the intervention group. Only Grossman et al. [40] described blinding of outcome assessors. All three authors described incomplete outcome data, including attrition rates, but only Grossman et al. [40] included this intention to treat and attrition data in the statistical analysis. There was no substantive evidence for selective outcome reporting in any of the studies, although Mills and Allen [41] omitted data on mood recording via Profile of Mood States (POMS), which is described elsewhere [43]. Overall, only the study by Grossman et al. [40] can be considered of high methodological quality (see Table 7).

**Table 7:** Risk of bias summary

	**Grossman et al. **[40]	**Mills and Allen **[41]	**Tavee et al. **[42]
**Random sequence generation** (selection bias)	Low	Unclear	NA
**Allocation concealment** (selection bias)	Low	Unclear	NA
**Blinding of assessors** (performance bias)	Low	Unclear	High
**Blinding of outcome assessment** (detection bias) (patient reported outcomes)	High	High	High
**Incomplete outcome data addressed** (attrition bias)	Low	Unclear	High
**Selective outcome reporting** (reporting bias)	Low	High	Unclear
**Other sources of bias** (ie baseline bias)	Low	Unclear	Unclear

1. Low = Low risk of bias; 2. Unclear = Unclear risk of bias; 3. High = High risk of bias; 4. NA = Not available.

## Discussion

This systematic review on the use of MBIs in people with MS identified three studies eligible for inclusion, which were varied in nature, with only one of the studies being adequately powered to calculate meaningful effect sizes (n = 150). Attrition rates across the studies were variable, and the reasons for this are unclear. The MBIs used in the studies were heterogenous. Two papers described a protocol comparable to MBSR, but stemming from Qi Gong practices; the other study closely resembled a more standardised version of MBSR [34]. Two of the interventions were applied in a hospital based group setting; one was individualised and delivered one to one in patients’ homes. There were a variety of measures of interest recorded. However, none of the studies focussed on our primary outcome of interest: perceived stress.

Results from the three studies are encouraging in the domains of mental health and HRQOL. Improvements in HRQOL, anxiety, depression, and fatigue remained statistically significant at six-month follow up in the Grossman et al. [40] study, albeit with diminished effect sizes; standing balance remained significantly improved at three-month follow up in the Mills and Allen [41] study. There were no adverse events reported.

### Strengths of this review

This review employed a rigorous methodological strategy to search and appraise the research literature involving MBIs in people with MS. Three reviewers were involved in the screening and appraisal of studies suitable for inclusion, with further discussion taking place, as required. Quality was assessed in accordance with the Cochrane Collaboration guidance.

### Limitations of this review

The fact that MBIs originate from ancient oriental traditions may bias the results, in that, due to resource constraints, our review was limited to papers published in English. The low methodological quality of two studies, and the overall heterogenous nature of the studies, precluded quantitative meta-analysis.

### Strengths and limitations of the included papers

Grossman et al. [40] produced a well designed RCT, with adequate numbers being included to allow power calculations. Their strict inclusion/exclusion criteria, and widely recognisable form of MBI, delivered by experienced, certified trainers, with pre-post and three-month follow up measures being collected, allows a degree of confidence when reviewing their findings. Mills and Allen [41] and Tavee et al. [42] conducted studies that were of lower methodological quality, with small sample sizes, and less well-defined intervention standards. There was no randomisation employed by Tavee et al. [42]. Comparing all three studies, there is considerable heterogeneity with respect to populations, interventions and outcome measures, and almost no meaningful information on the effects on different socioeconomic groups. Limited information was provided regarding different disease phenotypes and ‘stages’ of illness. Furthermore, little evidence is available about economic costs/benefits of the MBIs in this group, making drawing conclusions about any individual MBI technique in this population in general problematic. As such, these results should be treated with caution.

### Implications for research

Future studies of MBIs in people with MS should be of a larger scale, employing robust methodological techniques. They should examine physical and psychological measures; different disease phenotypes, at various defined stages of disease progression, of varied functional status; and should address important questions around feasibility, acceptability and appropriateness in diverse ethnic groups; as well as economic concerns such as health care utilisation and cost effectiveness. For specialist groups, such as people with MS, it may be worth examining whether having classes run by specialist trainers’ (i.e. health professionals) rather than ‘generic’ MBI trainers has any specific advantages/benefits.

Correlating findings with neuropsychological, biomarker and clinical imaging evidence would also be very informative. Given the widely varying attrition rates reported, qualitative research should also be employed to gather information on the broad acceptability of MBIs from the perspective of people with MS. Such research could also explore perceived stress and self-efficacy, as discussed previously.

### Implications for practice

MBIs may have utility in the MS clinical population, particularly for mental health conditions, such as anxiety and depression, as well as physical function. There is no overt evidence of harm.

## Conclusions

Although the evidence is limited, this review indicates that MBIs can hold benefit for people with MS, specifically in terms of quality of life, mental health, and some physical aspects of the condition. It is unclear at this time whether these results are generalisable to different ethnic groups; both genders; all age groups; different disease phenotypes; and diverse socio-economic groups. There is no evidence regarding health service utilisation costs. It also remains unclear what benefit MBIs may hold for people with more advanced MS. Further high quality studies are needed to clarify the feasibility, practicality, acceptability, health and psychosocial benefits of MBIs for people with MS.

## Competing interests

The authors declare that they have no competing interests.

## Author’s contributions

This systematic review was conceived by RS, SM, FM, ML and JB. RS registered this project with PROSPERO and took the lead for the review. RS conducted all database searches. RS, SB and JB carried out the screening of studies identified on database searching. RS authored the manuscript, whilst SM, FM, ML and JB all provided critical review and input into manuscript writing. All authors read and approved the final manuscript.

## Pre-publication history

The pre-publication history for this paper can be accessed here:

http://www.biomedcentral.com/1471-2377/14/15/prepub

## Supplementary Material

Additional file 1 Search history: OVIDsp - MEDLINE with Full Text 3/5/13 - pdf. Click here for file

Additional file 2 Excluded studies - pdf. Click here for file

## References

[B1] Franklin RJM Ffrench-Constant C Edgar JM Smith KJ Neuroprotection and repair in multiple sclerosis Nat Rev Neurol 2012 8 11 624 634 10.1038/nrneurol.2012.200 23026979

[B2] Scalfari A Neuhaus A Degenhardt A Rice GP Muraro PA Daumer M The natural history of multiple sclerosis, a geographically based study 10: relapses and long-term disability Brain 2010 133 7 1914 1929 10.1093/brain/awq118 20534650 PMC2892939

[B3] Chiaravalloti ND DeLuca J Cognitive impairment in multiple sclerosis The Lancet Neurol 2008 7 12 1139 1151 10.1016/S1474-4422(08)70259-X 19007738

[B4] Young CA Factors predisposing to the development of multiple sclerosis QJM 2011 104 5 383 386 10.1093/qjmed/hcr012 21382923

[B5] Dennison L Moss-Morris R Chalder T A review of psychological correlates of adjustment in patients with multiple sclerosis Clin Psychol Rev 2009 29 2 141 153 10.1016/j.cpr.2008.12.001 19167801

[B6] Damasceno A Von Glehn F Brandão CO Damasceno BP Cendes F Prognostic indicators for long-term disability in multiple sclerosis patients J Neurol Sci 2012 234 1 29 33 23073568 10.1016/j.jns.2012.09.020

[B7] Boland P Levack WM Hudson S Bell EM Coping with multiple sclerosis as a couple: ’peaks and troughs’-an interpretative phenomenological exploration Disabil Rehabil 2012 34 16 1367 1375 10.3109/09638288.2011.645115 22256892

[B8] McDonald WI Compston A Edan G Goodkin D Hartung H-P Lublin FD Recommended diagnostic criteria for multiple sclerosis: guidelines from the international panel on the diagnosis of multiple sclerosis Ann Neurol 2001 50 1 121 127 10.1002/ana.1032 11456302

[B9] Polman CH Reingold SC Banwell B Clanet M Cohen JA Filippi M Diagnostic criteria for multiple sclerosis: 2010 revisions to the McDonald criteria Ann Neurol 2011 69 2 292 302 10.1002/ana.22366 21387374 PMC3084507

[B10] McCrone P Heslin M Knapp M Bull P Thompson A Multiple Sclerosis in the UK Pharmacoecon 2008 26 10 847 860 10.2165/00019053-200826100-00005 18793032

[B11] Marrie R Horwitz R Cutter G Tyry T Campagnolo D Vollmer T Comorbidity, socioeconomic status and multiple sclerosis Mult Scler 2008 14 8 1091 1098 10.1177/1352458508092263 18728060

[B12] Dallmeijer AJ Beckerman H de Groot V van de Port IG Lankhorst GJ Dekker J Long-term effect of comorbidity on the course of physical functioning in patients after stroke and with multiple sclerosis J Rehabil Med 2009 41 5 322 326 10.2340/16501977-0335 19363563

[B13] Warren SA Turpin KV Pohar SL Jones CA Warren K Comorbidity and health-related quality of life in people with multiple sclerosis Int J of MS Care 2009 11 1 6 16 10.7224/1537-2073-11.1.6

[B14] Kang JH Chen YH Lin HC Comorbidities amongst patients with multiple sclerosis: a population‒based controlled study Eur J Neurol 2010 17 9 1215 1219 10.1111/j.1468-1331.2010.02971.x 20192982

[B15] Marrie R Horwitz R Cutter G Tyry T Campagnolo D Vollmer T The burden of mental comorbidity in multiple sclerosis: frequent, underdiagnosed, and undertreated Mult Scler 2009 15 3 385 392 10.1177/1352458508099477 19153176

[B16] Klevan G Jacobsen C Aarseth J Myhr KM Nyland H Glad S Health related quality of life in patients recently diagnosed with multiple sclerosis Acta Neurol Scand 2013 129 1 21 26 23772958 10.1111/ane.12142

[B17] McGuigan C Hutchinson M Unrecognised symptoms of depression in a community–based population with multiple sclerosis J neurol 2006 253 2 219 223 10.1007/s00415-005-0963-0 16177840

[B18] Chwastiak LA Ehde DM Psychiatric issues in multiple sclerosis Psychiatr Clin N Am 2007 30 4 803 817 10.1016/j.psc.2007.07.003 PMC2706287 17938046

[B19] Trojan DA Arnold D Collet J-P Shapiro S Bar-Or A Robinson A Fatigue in multiple sclerosis: association with disease-related, behavioural and psychosocial factors Mult Scler 2007 13 8 985 995 10.1177/1352458507077175 17468448

[B20] Bol Y Duits AA Hupperts RM Vlaeyen JW Verhey FR The psychology of fatigue in patients with multiple sclerosis: a review J Psychosom Res 2009 66 1 3 11 10.1016/j.jpsychores.2008.05.003 19073287

[B21] Mohr DC Hart SL Julian L Cox D Pelletier D Association between stressful life events and exacerbation in multiple sclerosis: a meta-analysis Bmj 2004 328 7442 731 10.1136/bmj.38041.724421.55 15033880 PMC381319

[B22] Mohr DC Lovera J Brown T Cohen B Neylan T Henry R A randomized trial of stress management for the prevention of new brain lesions in MS Neurology 2012 79 5 412 419 10.1212/WNL.0b013e3182616ff9 22786596 PMC3405245

[B23] Kern S Ziemssen T Review: Brain—immune communication psychoneuroimmunology of multiple sclerosis Mult Scler 2008 14 1 6 21 10.1177/1352458507079657 17881389

[B24] Thomas P Thomas S Hillier C Galvin K Baker R Psychological interventions for multiple sclerosis Cochrane Database Syst Rev 2006 1 1 53 10.1002/14651858.CD004431.pub2 PMC8406851 16437487

[B25] Kabat-Zinn J Wherever you go, there you are: Mindfulness meditation in everyday life: Hyperion 1994

[B26] Miller JJ Fletcher K Kabat-Zinn J Three-year follow-up and clinical implications of a mindfulness meditation-based stress reduction intervention in the treatment of anxiety disorders Gen Hosp Psychiatry 1995 17 3 192 200 10.1016/0163-8343(95)00025-M 7649463

[B27] Kabat-Zinn J Lipworth L Burncy R Sellers W Four-year follow-up of a meditation-based program for the self-regulation of chronic pain: treatment outcomes and compliance The Clinical Journal of Pain 1986 2 3 159 774 10.1097/00002508-198602030-00004

[B28] Piet J Hougaard E The effect of mindfulness-based cognitive therapy for prevention of relapse in recurrent major depressive disorder: a systematic review and meta-analysis Clin Psychol Rev 2011 31 6 1032 1040 10.1016/j.cpr.2011.05.002 21802618

[B29] Lawrence M Booth J Mercer S Crawford E A systematic review of the benefits of mindfulness‒based interventions following transient ischemic attack and stroke Int J Stroke 2013 8 6 465 474 10.1111/ijs.12135 23879751

[B30] Robinson FP Mathews HL Witek-Janusek L Psycho-endocrine-immune response to mindfulness-based stress reduction in individuals infected with the human immunodeficiency virus: a quasiexperimental study The JAltern & Complement Med 2003 9 5 683 694 10.1089/107555303322524535 14629846

[B31] Davidson RJ Kabat-Zinn J Schumacher J Rosenkranz M Muller D Santorelli SF Alterations in brain and immune function produced by mindfulness meditation Psychosom Med 2003 65 4 564 570 10.1097/01.PSY.0000077505.67574.E3 12883106

[B32] Carlson LE Speca M Patel KD Goodey E Mindfulness-based stress reduction in relation to quality of life, mood, symptoms of stress and levels of cortisol, dehydroepiandrosterone sulfate (DHEAS) and melatonin in breast and prostate cancer outpatients Psychoneuroendocrinology 2004 29 4 448 474 10.1016/S0306-4530(03)00054-4 14749092

[B33] Hölzel BK Lazar SW Gard T Schuman-Olivier Z Vago DR Ott U How does mindfulness meditation work? Proposing mechanisms of action from a conceptual and neural perspective Perspect Psychol Sci 2011 6 6 537 559 10.1177/1745691611419671 26168376

[B34] Kabat-Zinn J Full catastrophe living: Using the wisdom of your body and mind to face stress, pain, and illness: Delta 2009

[B35] Teasdale JD Segal ZV Williams JMG Ridgeway VA Soulsby JM Lau MA Prevention of relapse/recurrence in major depression by mindfulness-based cognitive therapy J Consult Clin Psychol 2000 68 4 615 10965637 10.1037//0022-006x.68.4.615

[B36] Kahl KG Winter L Schweiger U The third wave of cognitive behavioural therapies: what is new and what is effective? Curr Opin Psychiatry 2012 25 6 522 528 10.1097/YCO.0b013e328358e531 10.1097/YCO.0b013e328358e531 22992547

[B37] Richardson WS Wilson MC Nishikawa J Hayward RS The well-built clinical question: a key to evidence-based decisions ACP J Club 1995 123 3 A12 A13 7582737 10.7326/ACPJC-1995-123-3-A12

[B38] Reviews UoYCf D Akers J Systematic reviews: CRD’s guidance for undertaking reviews in health care: Centre for Reviews and Dissemination 2009

[B39] Higgins JP Altman DG Gøtzsche PC Jüni P Moher D Oxman AD The Cochrane Collaboration’s tool for assessing risk of bias in randomised trials BMJ: British Medical Journal 2011 343 d5928 10.1136/bmj.d5928 PMC3196245 22008217

[B40] Grossman P Kappos L Gensicke H D’Souza M Mohr DC Penner IK MS quality of life, depression, and fatigue improve after mindfulness training: a randomized trial Neurology 2010 75 13 1141 1149 10.1212/WNL.0b013e3181f4d80d 20876468 PMC3463050

[B41] Mills N Allen J Mindfulness of movement as a coping strategy in multiple sclerosis: a pilot study Gen Hosp Psychiatry 2000 22 6 425 431 10.1016/S0163-8343(00)00100-6 11072058

[B42] Tavee J Rensel M Pope Planchon S Stone L Effects of meditation on pain and quality of life in multiple sclerosis and polyneuropathy: a controlled study Int J MS Care 2011 13 S2 163 168 24453721 10.7224/1537-2073-13.4.163 PMC3882962

[B43] Mills N Allen J Carey-Morgan S Does Tai Chi/Qi Gong help patients with multiple sclerosis? J Bodyw Mov Ther 2000 4 1 39 48 10.1054/jbmt.1999.0139

