# Supplementary material for: Mindfulness based interventions in multiple sclerosis - a systematic review
Source: BMC Neurol. 2014 Jan 17;14:15. doi: 10.1186/1471-2377-14-15 (PMC3900731; doi:10.1186/1471-2377-14-15)
Supplement: Additional file 1 — Search history: OVIDsp - MEDLINE with Full Text 3/5/13 - pdf. [file 1471-2377-14-15-S1.pdf]

Additional file 1: Search history: OVIDsp - MEDLINE with Full Text 3/5/13

S1. exp multiple sclerosis/ or exp multiple sclerosis, chronic progressive/ or exp multiple sclerosis, relapsin-remitting/ or exp neuromyelitis optica/

S2. limit S1 to (english language and humans and yr=”1980-current”

S3. ("disseminated sclerosis" or "demyelinating disease" or devic or "acute disseminated encephalomyelitis" or "multiple sclerosis" or "neuromyelitis optica" or "optic neuritis" or "transverse myelitis").mp. [mp=title, abstract, original title, name of substance word, subject heading word, keyword heading word, protocol supplementary concept, rare disease supplementary concept, unique identifier]

S4. limit S3 to (english language and humans and yr="1980 -Current")

S5. exp Meditation/

S6. limit S5 to (english language and humans and yr="1980 -Current")

S7. ("breathing exercise$" or "breathing technique$" or mindful$ or meditat$ or relaxation or vipassana or yog$).mp. [mp=title, abstract, original title, name of substance word, subject heading word, keyword heading word, protocol supplementary concept, rare disease supplementary concept, unique identifier]

S8. limit S7 to (english language and humans and yr="1980 -Current")

S9. S2 or S4

S10. S6 or S8

S11. S9 and S10

Articles: 278
