# Supplementary material for: Mindfulness based interventions in multiple sclerosis - a systematic review
Source: BMC Neurol. 2014 Jan 17;14:15. doi: 10.1186/1471-2377-14-15 (PMC3900731; doi:10.1186/1471-2377-14-15)
Supplement: Additional file 2 — Excluded studies - pdf. [file 1471-2377-14-15-S2.pdf]

Additional file 2: Excluded studies - pdf

Author (Study title)	Factors necessitating exclusion
**Ahmadi et al. (2010)** The Effects of a Yoga Intervention on Balance, Speed and Endurance of Walking, Fatigue and Quality of Life in People with Multiple Sclerosis	Yoga intervention. No emphasis on Mindfulness described.
**Doulatabad et al. (2013)** The effects of pranayama, hatha and raja yoga on physical pain and the quality of life of women with multiple sclerosis	Yoga intervention. No full text available.
**Garrett et al. (2010)** Exercise in the community for people with multiple sclerosis—a follow-up of people with minimal gait impairment	Yoga intervention. No emphasis on Mindfulness described.
**Garrett et al. (2012)** Exercise in the community for people with minimal gait impairment due to MS: an assessor-blind randomized controlled trial	As Garrett et al. (2010). Study elaboration.
**Granmayeh et al. (2012)** The effects of Mindfulness-based Stress Reduction programme on physical symptoms, quality of life, and mental health in patients with multiple sclerosis	Full paper unavailable in English.
**Hankin (2010)** Mindfulness based stress reduction in couples facing multiple sclerosis: Impact on self reported anxiety and uncertainty	Uncontrolled trial.
**Jajvandian et al. (2011)** Influence of 6-week yoga on depression and fatigue in patients with multiple sclerosis, North Khorasan, northeastern Iran	Full paper unavailable in English.
**Nordin et al. (2012)** Cognitive behavioural therapy in multiple sclerosis: a randomized controlled pilot study of acceptance and commitment therapy	Acceptance and Commitment Intervention.
**Oken et al. (2004)** Randomized controlled trial of yoga and exercise in multiple sclerosis	Yoga intervention. No emphasis on Mindfulness described.
**Pritchard et al. (2010)** Impact of Integrative Restoration (iRest) Meditation on Perceived Stress Levels in Multiple Sclerosis and Cancer Outpatients	Yoga intervention. No emphasis on Mindfulness described.
**Velikonja et al. (2010)** Influence of sports climbing and yoga on spasticity, cognitive function, mood and fatigue in patients with multiple sclerosis	Yoga intervention. No emphasis on Mindfulness described.
